# BioShaDock: a community driven bioinformatics shared Docker-based tools registry

**DOI:** 10.12688/f1000research.7536.1

**Published:** 2015-12-14

**Authors:** François Moreews, Olivier Sallou, Hervé Ménager, Yvan Le bras, Cyril Monjeaud, Christophe Blanchet, Olivier Collin

**Affiliations:** 1 Genscale team, IRISA, Rennes, France; 2 Genouest Bioinformatics Facility, University of Rennes 1/IRISA, Rennes, France; 3 Centre d’Informatique pour la Biologie, C3BI, Institut Pasteur, Paris, France; 4 French Institute of Bioinformatics, CNRS IFB-Core, Gif-sur-Yvette, France

**Keywords:** bioinformatics, docker, container, deployment, interoperability, maintainability, community driven registry

## Abstract

Linux container technologies, as represented by Docker, provide an alternative to complex and time-consuming installation processes needed for scientiﬁc software. The ease of deployment and the process isolation they enable, as well as the reproducibility they permit across environments and versions, are among the qualities that make them interesting candidates for the construction of bioinformatic infrastructures, at any scale from single workstations to high throughput computing architectures. The Docker Hub is a public registry which can be used to distribute bioinformatic software as Docker images. However, its lack of curation and its genericity make it difﬁcult for a bioinformatics user to ﬁnd the most appropriate images needed. BioShaDock is a bioinformatics-focused Docker registry, which provides a local and fully controlled environment to build and publish bioinformatic software as portable Docker images. It provides a number of improvements over the base Docker registry on authentication and permissions management, that enable its integration in existing bioinformatic infrastructures such as computing platforms. The metadata associated with the registered images are domain-centric, including for instance concepts deﬁned in the EDAM ontology, a shared and structured vocabulary of commonly used terms in bioinformatics. The registry also includes user deﬁned tags to facilitate its discovery, as well as a link to the tool description in the ELIXIR registry if it already exists. If it does not, the BioShaDock registry will synchronize with the registry to create a new description in the Elixir registry, based on the BioShaDock entry metadata. This link will help users get more information on the tool such as its EDAM operations, input and output types. This allows integration with the ELIXIR Tools and Data Services Registry, thus providing the appropriate visibility of such images to the bioinformatics community.

## Introduction

The life sciences are becoming more and more digital and nowadays data analysis methods represent a key factor of the discovery process. In the case of bioinformatics, software is widely provided by the research community. Developers favor open source approaches and many software tools are available online. It is commonly agreed that such a distributed and free creation process accelerates discoveries in the life sciences ^1, 2^. However, this view must be nuanced, as multiple factors still hinder the discovery, integration, and maintenance of these software tools.

First, domains such as genomics, where technological innovation leads to a exponential growth of data to analyse, also generate an ever-increasing number of new software methods. However, the discovery of new interesting tools by potential users remains limited by unstructured descriptions, lack of metadata and deprecated source codes. In this context, dedicated search engines like the ELIXIR Tools and Data Services Registry ^3, 4^ (hereafter referred as the "ELIXIR registry") have emerged as a potential solution to search, find and locate available and maintained tools.

Secondly, the implementation methods of bioinformatic software are heterogeneous and their deployment requires multiple technical skills. The **installation process** is therefore expensive, in terms of human resources. It is worth recalling that the cost in supporting operating systems and hardware diversity can be high, the code compilation process is error prone and the required software dependencies are often conflicting with installed libraries. Consequently, the audience of a software can be limited to highly motivated and technical users or large bioinformatics facilities. The recent development of user-friendly data analysis environments like Galaxy ^5^ ease access for biologists and bio-analysts to bioinformatic tools. These software workbenches provide a generic web user interface for command line based scientific applications, but do not solve the tools’ deployment issue. Even if the task can be submitted inside a container, it is the tool designer’s responsibility to provide a readily deployable component ^6^ and the proportion of container based components in repositories such as the Galaxy Toolsheds ^7^ is currently low.

Finally, traditional academic publishing and funding processes emphasize the production of software with short-term goals, these being the publication of the method and/or results. Such an environment does not favor a software engineering-oriented approach to software development ^8^, and this affects directly the portability and maintainability of the software products ^9^. This in turn impacts the reproducibility of analyses, experiments or benchmarks described in published articles. However, even if various emerging initiatives are developing frameworks ^10– 12^ to enable a new kind of "executable format" of scientific publication, few journals have an innovative publishing policy that includes the long term storage of the source codes on a dedicated public web platform.

Nevertheless, today containerization brings new pragmatic solutions. Linux containers are a mature technology that has the potential to dramatically facilitate scientific software deployment and analysis reproducibility. Docker, one of the most popular container solutions ^13, 14^, is now used in a variety of computation environments, from commercial clouds ^15^ to clusters with dedicated middleware ^16^. It has been positively evaluated for data intensive computation, a recent study showing that the performance of bioinformatic workflows composed by medium or long running tasks are only very slightly affected by containerization ^17^.

Container technology has the potential to impact audiences, developers and end-users. In the scientific field, it can effectively improve reproducibility, ease deployment and facilitate the building of software collections and search engines dedicated to a specific scientific domain or topic.

For these reasons, we created the BioShaDock registry that promotes the use of container technologies in bioinformatics. The BioShaDock registry provides a web entry point to deploy, search and discover ready to use bioinformatics tools, encapsulated in Docker containers.

Future works will focus on better integration with domain-centric registries as well as bioinformatic integrated environments, to enable the seamless discovery, integration, and execution of the BioShaDock containers. Our project will also greatly benefit from discussions with other existing bioinformatic container initiatives.

## Methods

### Registration

**Figure 1.**
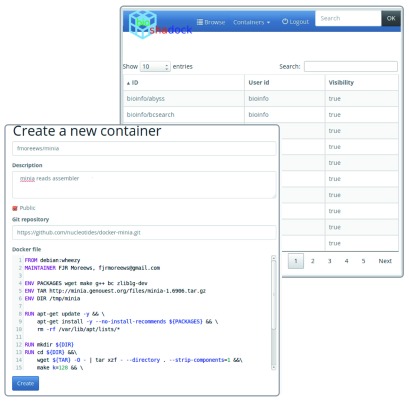
The BioShaDock web interface. The interface enables the creation of Dockerfiles and allows to search the repository using full text queries.

BioShaDock is a web server based system that allows the description, registration and automated building of Docker images ( Figure 1). These images are publicly available on the web server for search, download and execution. Users can authenticate using local LDAP or Google/GitHub credentials. LDAP users have the possibility to push new images. External users (Google, etc.) can request those privileges by contacting the support team. This mechanism allows non local users to have access to the registry to provide new tools while keeping a controlled access on the submission of new tools to the registry, where contributions are based on trust.

Once authenticated, the user can proceed to the **registration** of a Docker container. The information required includes:

- the **set of instructions to build the image**, i.e. the Dockerfile and the associated source code. These can be provided by pasting directly the Dockerfile contents in the web interface, by pointing to a Git repository that contains the Dockerfile and the source code, or by pointing to the source code repository and manually providing the Dockerfile. In the case of Git repository registration, it is also possible to configure the branch and location of the Dockerfile in the repository.
- **additional metadata** which is required to describe the contents of the image in scientific terms to its potential users. Such metadata includes for instance free tags, as well as EDAM ^18^ terms.

**Figure 2.**
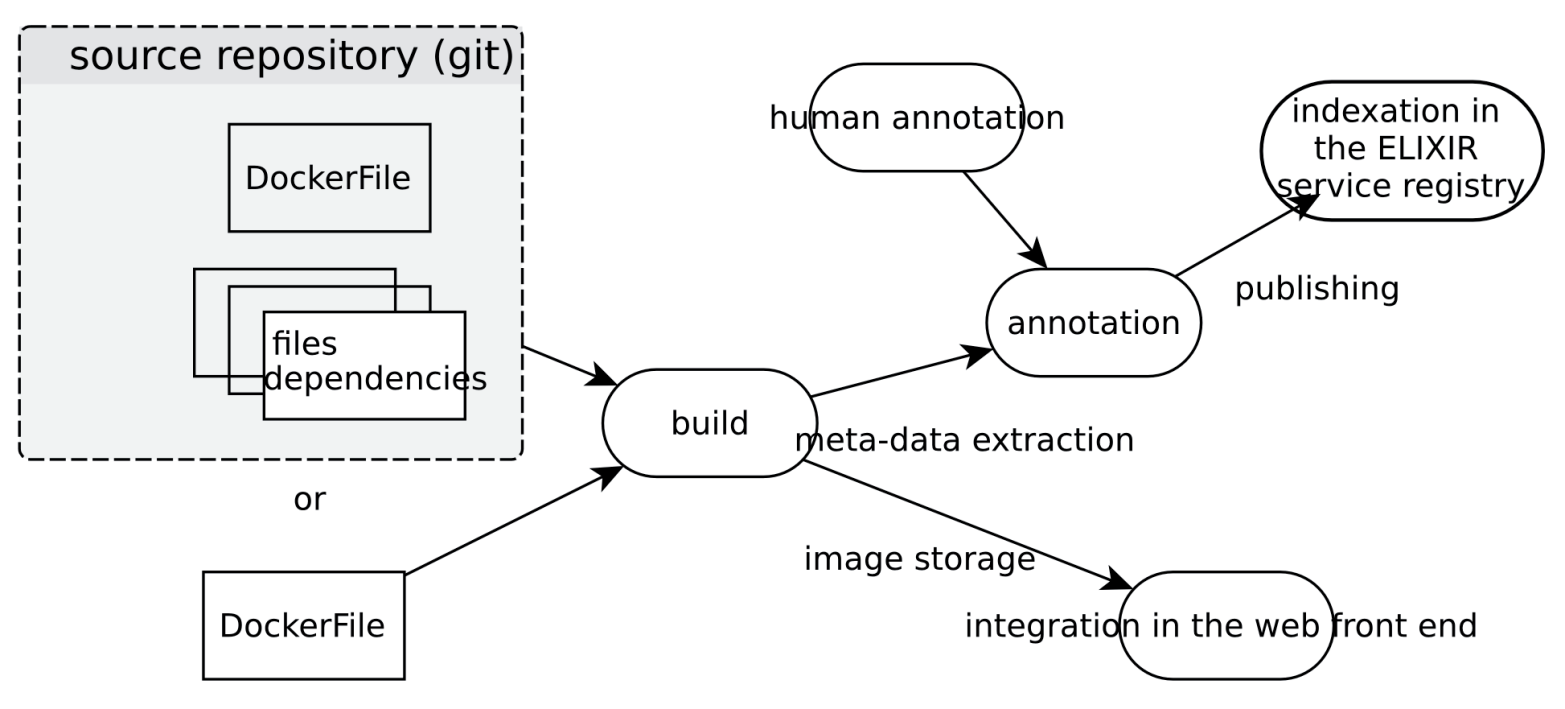
The BioShaDock Docker container processing steps.

Following the completion of container registration, the image construction and integration steps ( Figure 2) are automatically run on a dedicated server. The trigger of a new build is based on Dockerfile update or via a link (URL with an API Key), shown in the web interface when the user is the owner of the tool (created it). The creation of a tag on the image uses the same link mechanism. Such a link can be used directly (copy/paste in the brower) or via external tools or hooks (GitHub web hooks for example). The API also provides the possibility to trigger it manually, or to tag a container (i.e. set a version).

The Docker images, once built and stored in BioShaDock, can be registered in the ELIXIR registry (using some LABEL metadata in the Dockerfile). It is also possible to add a link to an existing ELIXIR registry entry. By linking its contents to and from the ELIXIR registry, BioShaDock enables the discovery of Docker images from a more generic system where users might look for a given software without specifically searching for container solutions. It hence maximizes the visibility of its images and contributes to better software dissemination.

### Search and execution

**Listing 1. An example of Docker image command line invocation using BioShaDock. After an automatic download, the container is executed. Here, the program BWA is called by default.**



                        sudo docker run docker-registry.genouest.org/bioinfo/\
bwa
Unable to find image \
’docker-registry.genouest.org/bioinfo/bwa:latest’\
locally
latest: Pulling from bioinfo/bwa
[...]
Status: Downloaded newer image for \
docker-registry.genouest.org/bioinfo/bwa:latest
Program: bwa (alignment via Burrows-Wheeler \ transformation)
Version: 0.7.5a-r405
[...]

                    


**Figure 3.**
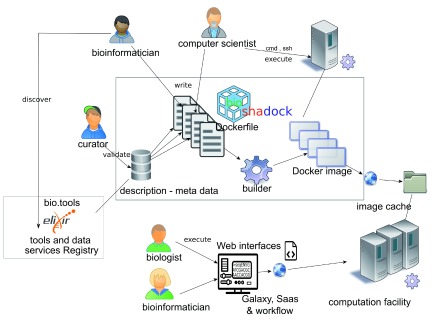
The BioShaDock use cases. The Docker repository acts as a platform that facilitates the dissemination of bioinformatics tools by providing ready to use Docker images.

The images provided by BioShaDock can be executed in various ways ( Figure 3):

• on a personal computer with a Linux system (Windows and Mac are supported with the Docker Toolbox), in a command line ( Listing 1), directly using Docker ^14^;
• on a cluster integrating a Docker scheduler front-end like GO-DOCKER (v1.0) ^16^;
• in any software implementing the CWL (Common Workflow Language) specification (draft 3) ^19, 20^ such as Arvados ^21^ or Rabix (v0.6.5) ^22^;
• in the D ^4^ workflow portal ^23^ (v0.6);
• in the Galaxy environment ^6^ (v15.10);
• in the cloud of the French Institute of Bioinformatics with the help of the Docker virtual machine image ^24^.

As an illustration, we created a set of Galaxy tool descriptors based on Docker images stored by BioShaDock ^25^ available in our Toolshed ^26^. Thus, the stacks RADSeq pipeline ^27^ is available as a Galaxy tool xml descriptor ^28^ that calls a container stored in BioShaDock ^29^.

### Implementation

**Listing 2. A container ’Dockerfile’ that defines the automated image build process. The LABEL instructions represent metadata.**



                        LABEL  name="Emboss"
LABEL  homepage="http://emboss.sourceforge.net/"
LABEL  resourceType="Tool"
LABEL  interfaceType="Command line"
LABEL  description="The European Molecular \
  Biology Open Software Suite"
LABEL  topic="Data processing and validation"
#EDAM operation
LABEL  functionName="Sequence processing"
FROM biodckr/biodocker:latest
USER root
# Install EMBOSS package
RUN apt-get update && \
    apt-get install -y \
      emboss=6.6.0-1 && \
    apt-get clean && \
    apt-get purge && \
    rm -rf /var/lib/apt/lists/* /tmp/* /var/tmp/*

USER biodocker
WORKDIR /data
CMD ["embossdata"]
MAINTAINER Adam Smith <asmithswx@cnrs.fr>
                    


BioShaDock is a web application written in python (>=2.7). It manages the container’s build and metadata. It is also in charge of authenticating the user against a local Docker registry and authorizing the user to push or pull a container according to their role (admin, editor, etc.) or rights. A user can give other users access to their repository for collaborative work in the edition page of the tool. Collaborators can have read only (for private repositories) or read/write access to the tool. The backend is based on a local instance of a Docker registry.

A script extracts the metadata written by the image’s maintainer ( Listing 2).

**Listing 3. An XML container metadata description generated from the LABEL instructions by BioShaDock and used to publish the container metadata in bio.tools, the ELIXIR registry.**



                         <?xml version="1.0" encoding="UTF-8"?>
<resources xmlns="http://bio.tools">
 <resource>
  <name>ngs_multi_vendor_read_corrector</name>
  <homepage>http://resourcename.org</homepage>
  <resourceType>Tool</resourceType>
  <interface>
   <interfaceType>Command line</interfaceType>
   </interface>
  <description>
   software analysis package specially developed for the needs of the molecular biology user community
  </description>
  <topic uri="http://edamontology.org/topic_0220"> Data processing and validation
  </topic>
  <function>
   <functionName uri="http://edamontology.org/operation_2446">
    Sequence processing
   </functionName>
   </function>
   <contact>
   <contactEmail>
    asmithswx@cnrs.fr
   </contactEmail>
  </contact>
 </resource>
</resources>
                    


Then, an integrated REST python client (v1.0) manages the container indexation in bio.tools ( Listing 3). The first version of the registry integrates 80 Docker images that are versioned and can be re-built when the sources are updated. A REST API enables programmatic interaction with the server. For example, it can be used by external tools to extract the list of available images for job submissions. GO-DOCKER (v1.0) and the D ^4^ workflow portal (v0.6) integrate this feature. The access to the images is public. To ensure the quality of available images, BioShaDock manages the authentication and ACL (access control list) to restrict the creation and update of its images to identified trustful contributors. The current implementation (v1.0) enables authentication using LDAP, Google or GitHub.

## Discussion

The aim of BioShaDock is to contribute to the aggregation and standardization of bioinformatic tools and utilities. Maintaining ready to use validated and versioned software is key in ensuring the reproducibility needed in an open science approach.

Thereby, the creation of a collection of tools embedded in Docker containers, as provided by BioShaDock, is a pragmatic solution to this major bottleneck.

A number of other projects also focus on the provision of bioinformatic Docker images. BioDocker ^30^ is a community based initiative to encourage the use of Docker images in bioinformatics. A GitHub repository stores a list of Dockerfiles that define the construction of images for the corresponding bioinformatic tool, with an open yet controlled contribution mechanism. Bioboxes ^31^ is an open source project that defines guidelines to build bioinformatic tool images using compatible interfaces for images which perform the same task, independent of the underlying tool, hence favoring interoperability between tools. It is therefore, among other characteristics, very well suited to automate tool and pipeline benchmarks. It has been applied to the assessment of different types of NGS data processing methods that concern assembly software as well as metagenomics tool. Dockstore ^32^ is an open platform that enables the registration of Docker images described using CWL. It integrates with a number of external services for source code and image hosting, and focuses on the provision of images that can be integrated in CWL-ready environments. BioShaDock shares with these existing efforts the use of Docker as a container technology to facilitate the distribution and integration of bioinformatic tools. However, none of these systems are designed to provide local image building and storage options. Furthermore, we believe the integration of BioShaDock with external domain-centric and platform-agnostic registries such as the ELIXIR registry will significantly raise the visibility of both the images provided and the container technology itself to the community of bioinformatic tool users. Because the files that describe the image building process (Dockerfiles) are usually freely available online, the interoperability issues between Docker registry initiatives are potentially very limited.

## Conclusions

Computer scientists and bioinformaticians can more easily disseminate their programs and find potential users using a dedicated domain-centric Docker registry. There is a wide range of perspective uses for container registries in bioinformatics: repositories managed at a community level, based on tools embedded in containers, promote the ability to exchange and replicate data analyses.

In addition, the association between workflow models, data references and containerized tools could lead to the creation of interoperable and ready to use analysis components and pipeline collections maintained by many contributors. The development of such specifications is already in progress as illustrated by the CWL (Common Workflow Language) ^20^ and the A-SCDFM (Autonomous Semi-Concrete Data Flow Model) ^33^ portable workflow formats that are natively compatible with containers. In this case, the integration of programs in a container registry like BioShaDock and the formalization of the data processing following one of these new portable workflow specifications could simplify the creation of reproducible benchmarks, teaching material, demos and the production of use case prototypes. It could also be used by article reviewers to quickly evaluate a software.

The spread of container usage in the bioinformatics community and their indexing in repositories can be a solution to capture and share a large collection of data analysis methods. A wide set of bioinformatics components available on demand could induce better data analysis by simplifying tests and benchmarks.

## Software availability

### Server

• BioShaDock registry: https://docker-ui.genouest.org
• BioShaDock home page: http://bioshadock.genouest.org

### Source code

• BioShaDock client and tools: https://github.com/fjrmoreews/bioshadock_client
• BioShaDock local server: https://bitbucket.org/osallou/bioshadock
• Archived source code at the time of publication (client): https://zenodo.org/record/34588 ^34^
• Archived source code at the time of publication (server): https://zenodo.org/record/34587 ^35^

### License

Apache 2.0

## Supplementary material

BioShaDock API documentation:

http://www.genouest.org/api/bioshadock-api
