# Supplementary material for: BioShaDock: a community driven bioinformatics shared Docker-based tools registry
Source: F1000Res. 2015 Dec 14;4:1443. [Version 1] doi: 10.12688/f1000research.7536.1 (PMC4743153; doi:10.12688/f1000research.7536.1)
Supplement: Peer review history

# Peer review: Referee response for version 1

**DOI:** 10.5256/f1000research.8115.r11546

**Published:** 2016-02-01

**Authors:** Björn A Grüning

**Affiliations:** 1 Department of Computer Science, University of Freiburg, Freiburg, Germany

This article describes very well the current state of bioinformatics Linux container adoption and arising problems. It offers solutions to these and also describes real-world use-cases with an existing integration into systems like Galaxy. Especially interesting is the rich annotation system, that involves ELIXIR ontologies as well as the ELIXIR registry.

This is needed and a big step forward.

Personally, I would like to see stronger collaborations between the mentioned other registry and Docker-build projects. I still feel we have a lot of redundant work inside of the bioinformatics community. For example I think it would be relatively easy to configure travis in biodocker to push automatically into BioShaDock, if biodocker counts as trusted partner. On the other hand biodocker can profit largely by the rich annotation system.

The manuscript is well written and I would encourage everyone to participate in this project. I certainly will.

I have read this submission. I believe that I have an appropriate level of expertise to confirm that it is of an acceptable scientific standard.

# Peer review: Referee response for version 1

**DOI:** 10.5256/f1000research.8115.r11548

**Published:** 2015-12-15

**Authors:** Rodrigo Lopez

**Affiliations:** 1 Wellcome Trust Genome Campus, European Bioinformatics Institute, Cambridge, UK

The article by Moreews *et al. *describes a registry of bioinformatic tools images that are portable using Docker technology. The manuscript is well written and describes well the aims of the BioShaDock registry and it's possible interactions with the ELIXIR Tools and Data Services Registry as the means to find Docker containers in the wild. As pointed out in the abstract, other Docker registries exists, such as Docket HUB, but lack of curation and user engagement hampers their progress. Furthermore,BioShaDock provides user management at a level required for ensuring that the interoperability between the registries,  images and local environments is secure, auditable and effective.

The article describes well the overheads associated with typical software installations and maintenance and presents a balanced view on the advantages of using Docker to manage this processes. 

Although not perhaps within the scope of this article, this reviewer feels it would be useful to inform the readership of other alternatives to Docker; e.g. Rocket, DrawBridge and LXD from Canonical and FlockPort, as it is clear that Docker is still maturing and it is certainly not the only container available today.

I have read this submission. I believe that I have an appropriate level of expertise to confirm that it is of an acceptable scientific standard.
